# Diffusion tensor imaging detects early brain microstructure changes before and after ventriculoperitoneal shunt in children with high intracranial pressure hydrocephalus

**DOI:** 10.1097/MD.0000000000005063

**Published:** 2016-10-21

**Authors:** Cailei Zhao, Yongxin Li, Weiguo Cao, Kui Xiang, Heye Zhang, Jian Yang, Yungen Gan

**Affiliations:** a Department of Radiology, The First Affiliated Hospital of Xi’an Jiaotong University, Xi’an; b Department of Radiology, Shenzhen Children's Hospital, Shenzhen; c The Key Laboratory of Biomedical Information Engineering, Ministry of Education, School of Life Science and Technology, Xi’an Jiaotong University, Xi’an; d Institute of Clinical Anatomy, School of Basic Medical Sciences, Southern Medical University, Guangzhou; e Shenzhen Institutes of Advanced Technology, Chinese Academy of Sciences, Shenzhen, China.

**Keywords:** apparent diffusion coefficients, diffusion tensor imaging, fractional anisotropy, high intracranial pressure hydrocephalus, ventriculoperitoneal shunt

## Abstract

To explore the use of diffusion tensor imaging (DTI) parameters in the quantitative assessment of early brain microstructure changes before and after ventriculoperitoneal shunt in children with high intracranial pressure hydrocephalus.

Ten patients with communicating hydrocephalus (age: 2–36 months) and 14 age-/gender-matched controls (age: 2–36 months) were enrolled in this study. All patients underwent the ventriculoperitoneal shunt procedure. The imaging data were collected before and 3 months after the operation. Regions of interests (ROIs) included the white matter near the frontal horn of the lateral ventricles (FHLV), the occipital horn of the lateral ventricles (OHLV), occipital subcortical (OS) area, frontal subcortical (FS) area, and thalamus. Fractional anisotropies (FA) and apparent diffusion coefficients (ADC) of the ROIs before and after ventriculoperitoneal shunt were compared between the patients and the controls.

Three months after surgery, the patients recovered from the surgery with ameliorated intracranial pressure and slight improvement of clinical intelligence scale and motor scale. Before ventriculoperitoneal shunt, the FA values (except the right FHLV) were significantly decreased and the ADC values were significantly increased in the patients with hydrocephalus, compared with the controls. After the ventriculoperitoneal shunt, the FA values in the FHLV and OHLV of the patients were similar to the controls, but the FA values in other ROIs were still significantly lower than controls. The ADC values in the FS and OS white matter areas of the patients were similar to the controls; however, the ADC values in other ROIs were still significantly higher in patients.

The increase of FA and the reduction in ADC in the ROIs preceded the clinical function improvement in patients with high intracranial pressure hydrocephalus and reflected the early changes in brain tissue microstructure, such as the compression of the white matter areas in the ROIs.

## 1 Introduction

Hydrocephalus causes severe brain tissue damage.^[1,2]^ The patients with hydrocephalus have progressive brain dysfunction due to ventricular enlargement and increased intracranial pressure. The main clinical treatment of hydrocephalus relies on surgical approaches. The surgical interventions can decrease ventricular enlargement and improve the cerebrospinal fluid (CSF) circulation, which can be detected by MRS (magnetic resonance spectrometer). However, the changes detected by MRS do not completely reflect the recovery from brain damage.^[3–5]^ Some of the clinical and experimental evidence suggests that the structural brain damages in patients with hydrocephalus are irreversible^[2,6,7]^; however, our previous clinical observations show different results. Here, we used clinical and radiographic parameters to evaluate the changes in the brain microstructure.

For patients with high intracranial pressure hydrocephalus, the major therapeutic purpose of ventriculoperitoneal shunt is to reduce intracranial pressure. The measurement of the intracranial pressure is essential in postoperative evaluations; however, the intracranial pressure monitoring is an invasive examination that may cause severe complications. Unfortunately, the noninvasive intracranial pressure monitoring methods are not yet well established.^[8,9]^ The evaluations of disease-related manifestations are subjective and the early changes in these indicators are subtle; therefore, these evaluations may not be able to provide accurate information. The tools that can accurately predict the long-term prognosis are still lacking; hence, it is necessary to develop a sensitive, noninvasive, and reliable biological indicator that can identify subtle neurophysiological changes before the deterioration or improvement of clinical symptoms. Conventional magnetic resonance (MR) can detect hydrocephalus caused brain structure abnormalities, but it lacks specificity in quantification of white matter damage. Diffusion tensor imaging (DTI) has been used to evaluate the changes in the brain structure, especially the integrity of the white matter.^[10–12]^ It has been proven to be sensitive to quantify the white matter injury and damage recovery.^[13,14]^ However, most of the studies have focused on the adult patients with normal intracranial pressure hydrocephalus, only very few studies have assessed the DTI changes in children with chronic hydrocephalus before and after surgery. Moreover, some of the studies have limited number of patients, variable postoperative assessment duration, or without standard Bayley or Wechsler scale scores.^[15–17]^ Studies show that different brain areas have different sensitivity in patients with hydrocephalus.^[16,17]^ The aim of this study was to use DTI to evaluate the early postoperative changes in the brain tissue microstructures at different brain regions in children with hydrocephalus.

The continuous liquid exchange between CSF and brain parenchyma is the main way of CSF production. Aquaporin-4 (AQP4) is distributed along the brain–liquid interfaces and is involved in CSF circulation.^[18]^ We chose the periventricular region and bilateral subcortical white matter areas as the regions of interests (ROIs) because these regions are the interfaces of CSF and brain parenchyma, the direct point of mechanical pressure, and the important pathway of CSF absorption into the brain parenchyma and blood flow.^[18,19]^ Although this is a retrospective study, we strictly selected enrolled patients with similar hydrocephalus and underwent similar surgical intervention and standardized timing and parameters of MR acquisition. Our goal is to use DTI to evaluate the early changes in the brain microenvironment in children with communicating hydrocephalus and to elucidate the mechanism of injury and recovery of the patients.

## 2 Materials and methods

### 2.1 Subjects

Ten continuous patients with communicating hydrocephalus were enrolled in Shenzhen Children's Hospital from 2013 to 2014—6 females and 4 males, aged 2 to 36 months, with a mean age of 15.6 ± 13.47 months. All patients underwent ventriculoperitoneal shunt treatment. The inclusion criteria of the patients were as follows: the head circumference progressively increased without medical history of acute intracranial hypertension; the clinical manifestations of high intracranial pressure, such as headache, vomiting, papilledema, and bulging fontanelle; communicating hydrocephalus confirmed by MR imaging and computed tomography or MR ventricular angiography without intracranial masses, and Evans index >0.33; and the mental and motor development scores were measured using Bayley or Wechsler (second edition) scales before and after surgery. The source and inclusion criteria of the controls were as follows: all of the children in the control group were treated with other diseases at the Shenzhen Children's Hospital; they received medical examination; they had normal development statues according to the World Health Organization (WHO) “Child Growth Standards (2006)”; they had normal nervous system development; and the illness does not affect the nervous system. The patient and control groups were matched 1:1 by age and gender. For patients with hydrocephalus, 6 cases had hydrocephalus after intracerebral hemorrhage and subarachnoid hemorrhage, and 4 cases had hydrocephalus after encephalitis.

All patients with hydrocephalus were scanned 2 times: first scan was done before ventriculoperitoneal shunt and second scan was done at 3 months after ventriculoperitoneal shunt. The controls were scanned 1 time. The study protocol was approved by the Ethics Committee of Shenzhen Children's Hospital. Families of the participants signed a written informed consent when enrolled into the study.

## 3 Methods

### 3.1 Image acquisition

Foam cushions were used to reduce head movements. The patients and the controls received chloral hydrate (0.5 mL/kg, the total volume was less than 10 mL) before scanning. Imaging data were collected using an 8-channel head coil on a 3T Siemens scanner (Skyra, Siemens, Erlangen, Germany) at the Shenzhen Children's Hospital (Shenzhen, China). The parameters for conventional axial T_1_WI and T_2_WI are as follows—T_1_WI: section thickness of 5 mm, spacing of 1 mm, repetition time (TR) of 2307 ms, and echo time (TE) of 10.6 ms; T_2_WI: fast spin echo sequence: section thickness of 5 mm, spacing of 1 mm, TR of 4000 ms, and TE of 103 ms; sagittal T_1_WI: section thickness of 5 mm, spacing of 1.5 mm, TR of 200 ms, and TE of 2.49 ms. The DTI protocol was using a spin echo planar image sequence with the following parameters: TR/TE = 6800/93 ms, field of view = 220 × 220 mm^2^, 40 axial slices, slice thickness = 2.5 mm, and in-plane resolution = 1.719 × 1.719 mm^2^. Diffusion weighing was isotropically distributed along 30 directions (b = 1000 s/mm^2^).

### 3.2 Imaging processing

**Figure 1:**
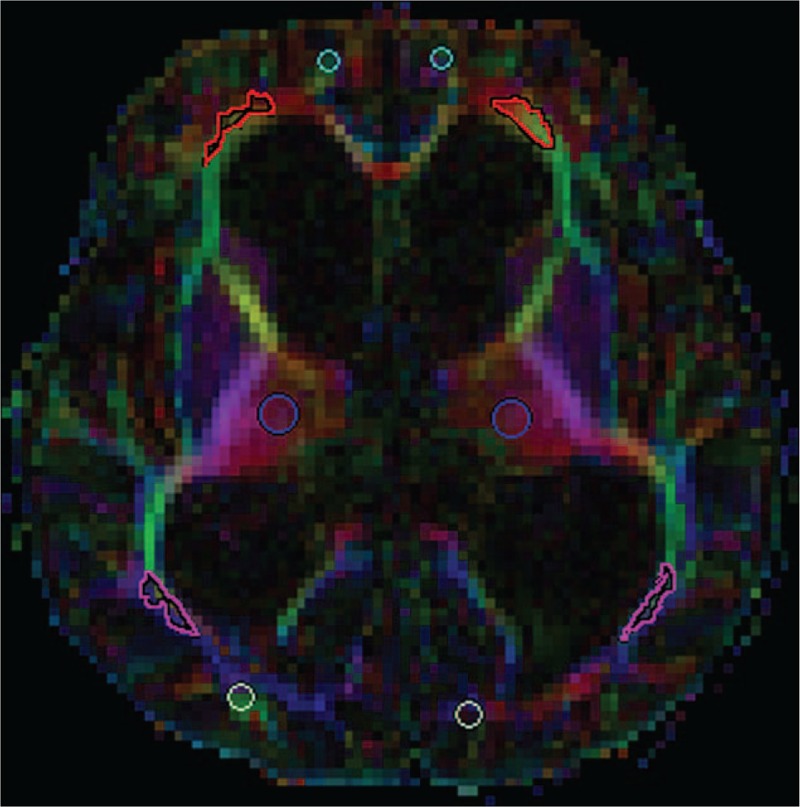
Diffusion tensor imaging maps demonstrating the location of the regions of interests. Light blue represents the frontal subcortical region; red represents the frontal horn of the lateral ventricle; blue represents the occipital horn of the lateral ventricle; rosiness represents thalamus and white represents occipital subcortical region.

The images were processed by an experienced radiologist blinded to the experiment using a syngo multimodality workplace. Tract-based spatial statistics could not be applied due to the significantly enlarged ventricles and severe brain tissue damages in patients with hydrocephalus. Therefore, ROIs were manually selected using a color-coded fractional anisotropies (FA) map. The ROI curve was placed on the same fiber bundle based on the color code, thereby reducing the error caused by the hydrocephalus-induced deformation of the periventricular structures. The ROIs were set as a sphere (20 mm^2^ in diameter) in the whiter matter (WM) nearby the FHLV, the OHLV, and thalamus. A sphere (5 mm^2^ in diameter) was used in the WM nearby occipital and frontal subcortical (FS) areas. The occipital and FS regions had small and irregular shapes; hence, we chose a relatively small area to keep the consistency among all of the patients. The locations of the ROIs were shown in Fig. 1. The mean FA and apparent diffusion coefficients (ADC) values of ROIs were compared between the patients and the normal controls.

### 3.3 Clinical assessments

The cognition and motor function of the patients and the controls were assessed by 1 certified pediatrician. Bayley scale was used for children with hydrocephalus younger than 30 months. Wechsler scale (second edition) was used for children with hydrocephalus older than 30 months. WHO “Child Growth Standards (2006)” was used for normal control group.

### 3.4 Statistical analysis

Statistical analysis was performed using SPSS16 (Stanford University, California, The United States) software. The ADC and FA values were inputted into the software. Two-tailed *t* test was used to compare FA and ADC values between the patient group and the control group both before and after ventriculoperitoneal shunt. Within the same group, paired *t* test was used to compare the changes pre- versus postshunt. The threshold for statistical significance was set as *P* < 0.05.

## 4 Results

### 4.1 The clinical assessments

The high intracranial pressure ameliorated in patients after ventriculoperitoneal shunt. The pump pressure was 40 to 150 mmH_2_O lower than the preoperative intracranial pressure. This pressure adjustment was based on the experience of the doctors. No complications occurred after surgery. Three months after surgery, the patients recovered from the impact of the surgery, clinical intelligence and motor scales or total intelligence quotient were slightly improved. The clinical intelligence scale and motor scale show various degrees of improvement, but the intelligence scale show better improvement than the motor scale (Table 1). Children in the control group had no neurological symptoms and showed normal development according to the WHO “Child Growth Standards (2006)”. MRI showed no intracranial lesion in the controls.

**Table 1:**
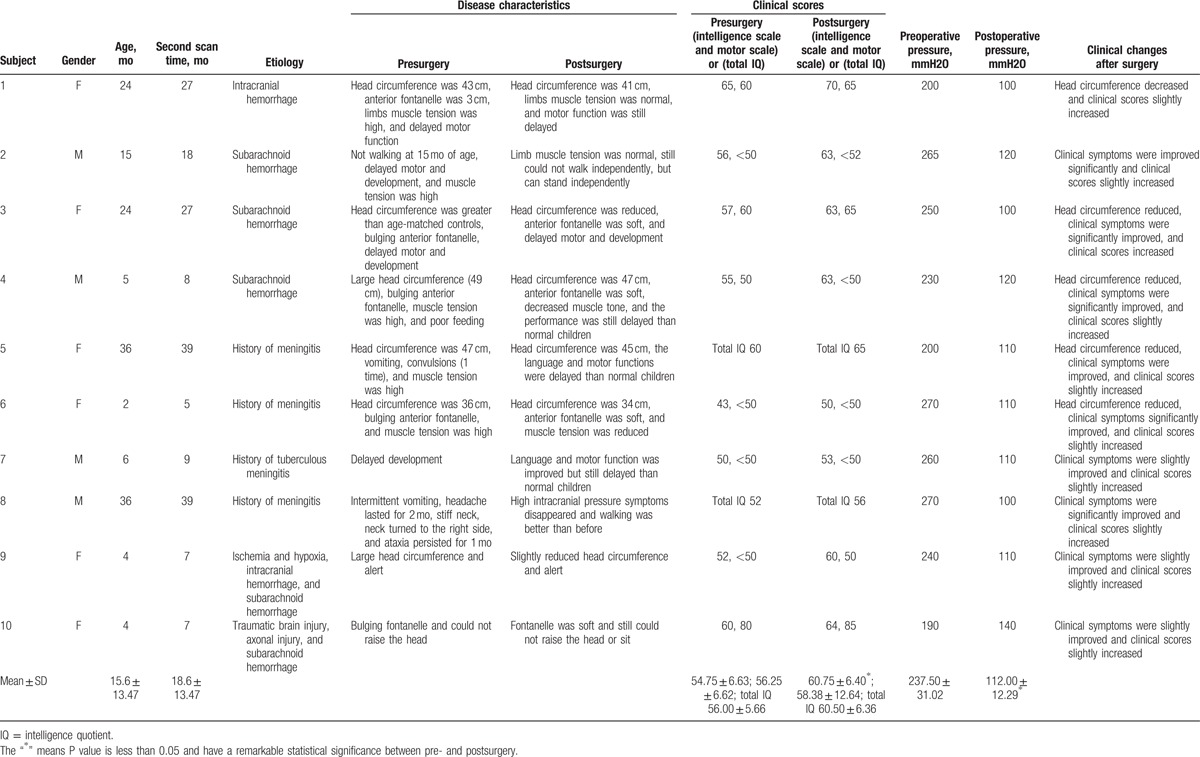
Patients’ information.

### 4.2 The changes in the FA values

**Figure 2:**
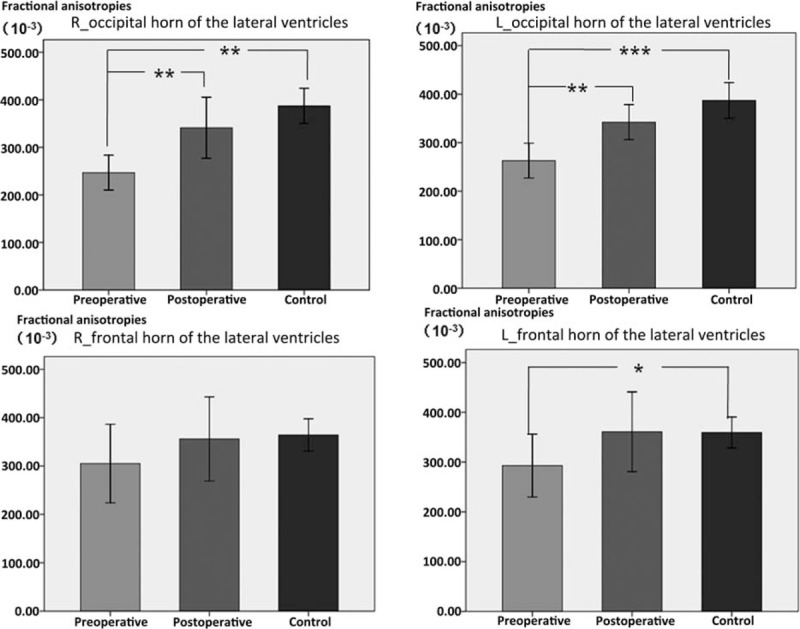
The mean fractional anisotropies (FA) values of the patient with high-pressure hydrocephalus and controls. The error bars represent the 95% confidence interval (CI) of the group means. Pre, preshunt; Post, postshunt; Con, control group. The FA values of the 4 regions, R-OHLV (occipital horn of the lateral ventricles), L-OHLV, R-FHLV (lateral ventricle), and L-FHLV, increased after treatment. The results of intergroup and intragroup statistical analysis are shown as follows: the FA values of R-OHLV were significantly different between the controls and the patients with hydrocephalus before treatment. However, the FA values of R-OHLV were similar between the controls and the patients after treatment. Moreover, the FA values of R-OHLV in the patients with hydrocephalus were significantly different before and after treatment. The FA values of L-OHLV were markedly different between the controls and the patients with hydrocephalus before treatment. However, the FA values of L-OHLV were similar between the controls and the patients after treatment. Moreover, the FA values of L-OHLV in the patients with hydrocephalus were significantly different before and after treatment. The FA values of R-FHLV were significantly different between the controls and the patients with hydrocephalus before treatment. However, the FA values of R-FHLV were similar between the controls and the patients after treatment. The FA values of R-FHLV in the patients with hydrocephalus were similar before and after treatment. The FA values of L-FHLV were significantly different between the controls and the patients with hydrocephalus before treatment. However, the FA values of L-FHLV were similar between the controls and the patients after treatment. The FA values of L-FHLV in the patients with hydrocephalus were similar before and after treatment. ^∗^*P* < 0.05; ^∗∗^*P* < 0.01; ^∗∗∗^*P* < 0.001.

We compared the FA values between the preoperative hydrocephalus patients and the control group. We found that there are statistical differences in FA values in the R-FS and L-FS regions (*P* = 0.008), the R-FHLV (*P* = 0.144), and L-FHLV (*P* = 0.046), the R-thalamus and L-thalamus (*P* = 0.001), the R-OHLV and L-OHLV (*P* = 0.000), the R-occipital (*P* = 0.000), and L-occipital areas (*P* = 0.002), compared with the control group (Fig. 2).

We then compared the FA values between the postoperative hydrocephalus patients and the control group. We found that *P* = 0.004 and *P* = 0.045 in the R-FS and L-FS regions, respectively; *P* = 0.777 and *P* = 0.640 in the R-FHLV and L-FHLV (Fig. 2), respectively; *P* = 0.007 and *P* = 0.006 in the R-thalamus and L-thalamus, respectively; *P* = 0.157 and *P* = 0.095 in the R-OHLV and L-OHLV (Fig. 2), respectively; and *P* = 0.013 and *P* = 0.021in the R-occipital and L-occipital regions, respectively, compared with the control group.

The FA values significantly decreased in most of the ROIs (expect of the right FHLV and the thalamus) in children with hydrocephalus before ventriculoperitoneal shunt, compared with the healthy controls (Fig. 2). There was no significant difference in the FA value in the right FHLV between the patients and the controls. However, the FA values in bilateral thalamus were significantly increased in the patients with hydrocephalus. The FA values significantly increased after the ventriculoperitoneal shunt in all ROIs (except thalamus) in patients with hydrocephalus. There was no significant difference in the FA values in bilateral OHLV and FHLV, and the left occipital lobe between the patients and the controls (Fig. 2). Although the FA values in bilateral thalamus decreased after ventriculoperitoneal shunt, they remained significantly higher in the patients with hydrocephalus. The FA values in other ROIs showed a trend toward normal values, but still significantly different from the controls (Table 2).

**Table 2:**
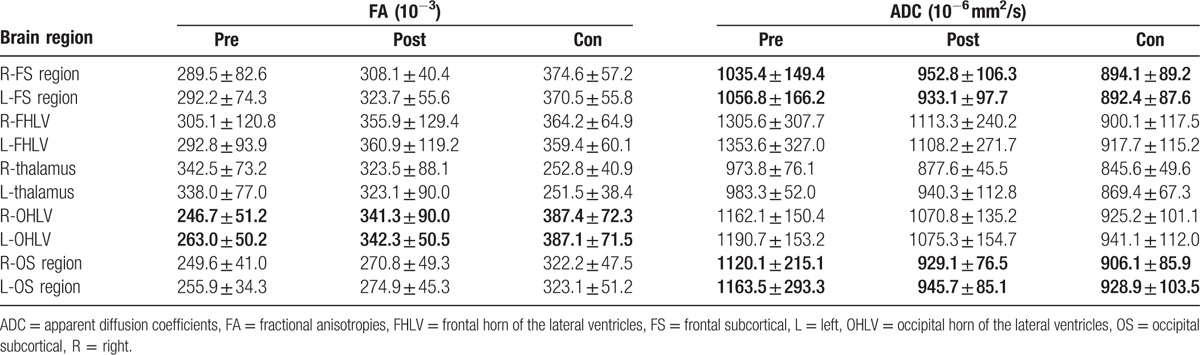
The changes in the FA and ADC before and after ventriculoperitoneal shunt.

### 4.3 The changes in the ADC values

**Figure 3:**
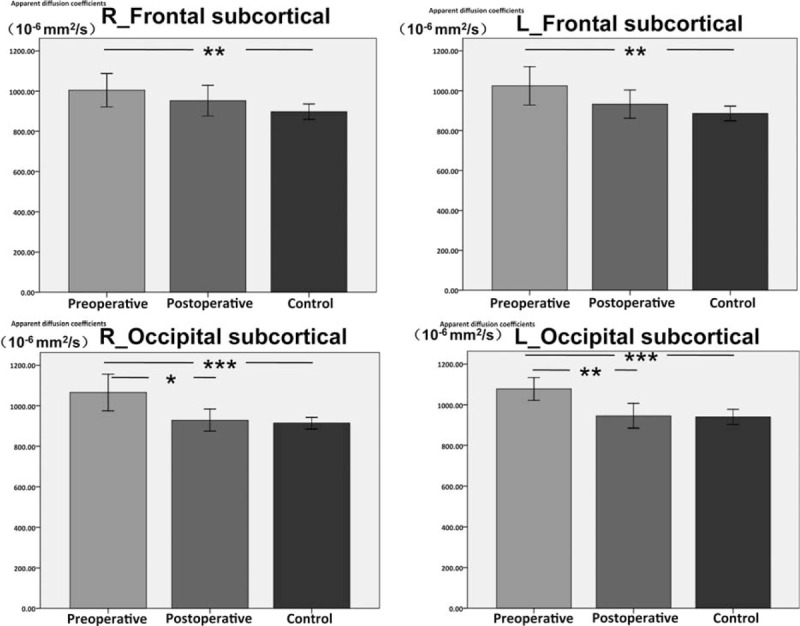
The mean apparent diffusion coefficients (ADC) values of the patient with high-pressure hydrocephalus and controls. The error bars represent the 95% CI of the group means. Pre, preshunt; Post, postshunt; Con, control group. The ADC values of the 4 regions, R-frontal subcortical (FS), L-FS , R-occipital subcortical (OS), and L-OS, decreased after treatment. The results of intergroup and intragroup statistical analysis are shown as follows: the ADC values of R-FS were significantly different between the controls and the patients with hydrocephalus before treatment. However, the ADC values of R-FS were similar between the controls and the patients after treatment. The ADC values of R-FS in the patients with hydrocephalus were similar before and after treatment. The ADC values of L-FS were significantly different between the controls and the patients with hydrocephalus before treatment. However, the ADC values of L-FS were similar between the controls and the patients after treatment. The ADC values of L-FS in the patients with hydrocephalus were similar before and after treatment. The ADC values of R-OS were markedly different between the controls and the patients with hydrocephalus before treatment. However, the ADC values of R-OS were similar between the controls and the patients after treatment. Moreover, the ADC values of R-OS in the patients with hydrocephalus were significantly different before and after treatment. The ADC values of L-OS were markedly different between the controls and the patients with hydrocephalus before treatment. However, the ADC values of L-OS were similar between the controls and the patients after treatment. Moreover, the ADC values of L-OS in the patients with hydrocephalus were significantly different before and after treatment. ^∗^*P* < 0.05; ^∗∗^*P* < 0.01; ^∗∗∗^*P* < 0.001.

We compared the ADC values between the preoperative hydrocephalus patients and the control group. We found that the *P* = 0.01 and *P* = 0.005 in the R-FS and L-FS regions (Fig. 3), respectively; *P* = 0.000 in the R-FHLV and L-FHLV; *P* = 0.000 in the R-thalamus and L-thalamus; *P* = 0.000 in the R-OHLV and L-OHLV; and *P* = 0.001 in the R-occipital and L-occipital regions (Fig. 3), compared with the control group.

We then compared the ADC values between the postoperative hydrocephalus patients and the control group. We found that *P* = 0.136 and *P* = 0.275 in the R-FS and L-FS regions (Fig. 3), respectively; *P* = 0.010 and *P* = 0.033 in the R-FHLV and L-FHLV, respectively; *P* = 0.154 and *P* = 0.050 in the R-thalamus and L-thalamus, respectively; *P* = 0.004 and *P* = 0.015 in the R-OHLV and L-OHLV, respectively; and *P* = 0.492 and *P* = 0.669 in the R-occipital and L-occipital regions (Fig. 3), respectively, compared with the control group.

The ADC values in all ROIs significantly increased in the children with hydrocephalus before ventriculoperitoneal shunt, compared with the healthy controls. After ventriculoperitoneal shunt surgery, the ADC values in the patients with hydrocephalus showed a trend toward normal values (Table 2). There was no significant difference in the bilateral FS and occipital subcortical (OS) regions and left thalamus between the patients and the normal controls (Fig. 3). There were significant changes in the ADC values in the FHLV, OHLV, and right thalamus, before and after ventriculoperitoneal shunt.

## 5 Discussion

At the 2008 hydrocephalus conference held in Hannover, Germany, Dr Harold proposed that hydrocephalus is due to dysregulation of the production and absorption of CSF, resulting in continued enlargement of the ventricles. The viscoelastic index of brain tissue is fixed and limited; therefore, the continuous enlargement of the ventricles compresses the whole brain tissues and intracranial lacunar. If left untreated, the enlarged ventricles will continuously compress the brain tissues.^[20]^ The researchers from the National Science Foundation proposed that the hydrocephalus is a devastating disorder.^[21]^ It will not heal automatically without treatment, and the white matter development in the patients will be significantly delayed than the normal controls.^[22]^ Therefore, surgical intervention is very important for brain tissue recovery. We chose the periventricular brain region as the ROI, because the periventricular region is the most affected area by increased intracranial pressure and ventricular enlargement. In this study, we compared the DTI characteristics pre- and postventriculoperitoneal shunt in the patients with hydrocephalus with the normal control group and analyzed the repairment of brain damage after ventriculoperitoneal shunt. All patients in this study underwent ventriculoperitoneal shunt. These patients had ameliorated intracranial pressure and exhibited slight improvement of clinical intelligence and motor function at 3 months postsurgery; however, these patients still showed delayed development than the age- and gender-matched controls. Some of the patients showed relatively small improvements, while some other patients did not show any improvement. We used DTI characteristics to quantify the subtle changes in the brain tissues. We demonstrate that the ADC values of the white matter areas in the bilateral frontal and occipital cortex were significantly reduced, while the FA values of the white matter around the lateral ventricles were significantly increased in the patients with hydrocephalus.

### 5.1 The changes in the FA values in patients with hydrocephalus

FA, one of the DTI characteristics, has been widely used in clinical practice and is the most sensitive indicator of the white matter integrity. The FA value is closely related to the myelin integrity, nerve fiber density, and orientation.^[10,23,24]^ In this study, we found that the FA values of ROIs before the ventriculoperitoneal shunt were substantially lower in the patients than the control group, which might reflect the demyelination of the white matter caused by enlarged ventricles and high intracranial pressure.^[25,26]^ The FA values significantly increased in the patients after surgery, which might be due to the reduced intracranial pressure, improved CSF circulation, and restored myelination.^[27]^ The result can be explained by the intelligence scale in Table 1. Postsurgery, the intelligence has a little increase. But the motor scale has no remarkable change, so using FA to explore the change in the early stage of postsurgery is more valuable. Studies show that the FA values of the white matter areas are often increased in the adult with normal pressure hydrocephalus and acute hydrocephalus. The white matter myelination has been completed in adults before the onset of hydrocephalus; therefore, the density of the white matter increases due to the compression of the tissues, resulting in the increased FA values.^[23,28,29]^ When the intracranial pressure decreased after surgery, the white matter density reduced, resulting in the decreased FA values. Therefore, the changes in the brain tissue microstructures are different in children and adults with hydrocephalus.

The patients in our study had an average age of 17 months, and the myelination process continues at this age bracket.^[30]^ The ventriculoperitoneal shunt ameliorates the intracranial pressure, which may improve the myelination and restore the white matter integrity, resulting in the increased FA value. Myelination proceeds in a posterior-to-anterior pattern during brain development. In this study, the FA values in the white matter areas of the bilateral OHLV showed the most significant changes and achieved the level of normal controls. Myelination in the thalamus begins at birth and is completed at 17 months of age.^[30]^ Therefore, the change in the FA values in the thalamus of the patients in our study showed similar patterns as the adult patients with hydrocephalus. In our study, the FA values in the thalamus increased before surgery due to the compression of the third ventricle. The compression of the third ventricle relived, and the nerve fiber density decreased after surgery, resulting in decreased FA values. The FA values in the patients were still significantly higher than the controls, which might be due to the incomplete brain tissue recovery from the third ventricle compression.

Clinical tests show that the intellectual scale improved better than the motor scale, which can be explained by the nerve fiber connectivity and function. The nerve fibers at the OHLV connect the Wernicke area and the Broca area, occipital and temporal lobes, and involved in cognitive function (language, visual spatial skills, attention, verbal, and visual memory).^[31]^ The FA value in this area showed the fastest recovery and returned to the normal control level in the patients with hydrocephalus; hence, the intelligence scale improved rapidly. In contrast, the posterior internal capsule is associated with motor function and had the slower recovery of microstructures; hence, the motor function of the patients with hydrocephalus did not significantly improve (score <50 points) at 3 months after surgery. These results further demonstrate that the increased intracranial pressure and the enlarged ventricle are the major factors that cause abnormal myelination; therefore, it is important to reduce intracranial pressure and prevent ventricle enlargement in children with hydrocephalus.^[15]^ Moreover, the white matter myelination, especially the OHLV, can be quickly restored in children. These results show that the white matter injury is reversible in children with hydrocephalus.

### 5.2 The changes in the ADC values in patients with hydrocephalus

ADC value is an indicator of the in vivo water molecule movement and has been widely used in the clinical practice.^[32]^ In the past studies, the CSF circulation has been monitored by intracranial tracer injection. However, toxicity and molecular size of the tracer hinder its application.^[33]^ The ADC value is an excellent indicator of water movement and is used for the evaluation of CSF circulation.^[18,32]^ In our study, the ADC values of all ROIs in the patients with hydrocephalus were significantly higher than the controls before surgery, indicating the excessive accumulation of interstitial fluid. Studies show that the blood–brain barrier within the brain parenchyma is the main place for the exchange of CSF.^[18,33,34]^ The impaired exchange between the interstitial fluid and CSF leads to the accumulation of CSF within the ventricular system and results in ventricular enlargement.

The ADC values in the white matters of the ROIs (bilateral FS and OS regions, FHLV and OHLV, and thalamus) significantly decreased after the surgery, indicating significantly reduced extracellular fluids. These results suggest that surgery reduced intracranial pressure, increased blood flow, decreased capillary pressure, increased the permeability of capillaries and brain parenchyma,^[35]^ and improved CSF circulation.

Our results show that the fluid exchange in the bilateral FS and OS white matter areas is similar to the normal control group. The ADC value at the periventricular region decreased after surgery, but has not reached the level of the control group, which might be due to the ependymal and subependymal astrocytes damage caused by the continuous ventricle enlargement.^[36–39]^ Moreover, the AQP4 located on the astrocytes plays crucial roles in the water transport,^[18,34]^ and the dysfunction of the AQP4 may cause abnormal water exchange. Our results indicate that ADC values of bilateral FS and OS white matter decreased quickly after surgery, which may reflect the early recovery of the brain tissue microstructure. We found that the ADC values rapidly decreased at 3 months postsurgery, which are different from the previous results found from adult patients. Previous studies show that the ADC values before and after surgery in the adults with normal pressure hydrocephalus are similar to the normal controls.^[11]^ In our pediatric patients, the brain water content is much higher than the adults, and the impaired water exchanges will have more significant impacts on the ADC values.

This study has some limitations. First, our patient sample size is small, and the pathology of injuries in each individual is variable. Therefore, we were not able to provide a classification. Second, we did not have the detailed records of clinical functional recovery scores; therefore, we did not analyze the correlation of the clinical functional recovery scores and the DTI parameters. In our future studies, we will recruit more patients, classify the injury pathology, collect complete clinical functional recovery scores, and analyze the correlation of the clinical functional recovery scores and the DTI parameters.

## 6 Conclusion

FA and ADC, the DTI characteristics, are valuable in the evaluation of early recovery in children with chronic hydrocephalus. FA and ADC can detect the early changes in the brain microstructures when there is little or no improvement of clinical intelligence and motor scales. The increased FA values indicate the improvement of the white matter integrity, while the decreased ADC values suggest improvement of CSF circulation. Therefore, FA and ADC values can be used as sensitive and noninvasive assessments of the early changes in the brain microenvironment.

## Acknowledgments

We thank the participants and their families for their participation in this study. We thank YG for his help and support.
